# Influence of data disclosures on physician decisions about off-label uses: findings from a qualitative study

**DOI:** 10.1186/s12875-022-01666-2

**Published:** 2022-04-19

**Authors:** Melanie C. Chansky, Simani M. Price, Kathryn J. Aikin, Amie C. O’Donoghue

**Affiliations:** 1 Westat, Maryland USA; 2 U.S. Food and Drug Administration, Maryland, USA

## Abstract

**Background:**

Prescribing approved products for unapproved uses (off-label use) is not uncommon among physicians in certain medical specialties. Available evidence about an off-label use – both supportive and unsupportive – can influence prescribers’ decisions about a drug’s appropriateness for a particular case. The objectives of this study were: (1) to examine physician perceptions about off-label uses generally, including their awareness of unsupportive data; and (2) to explore the influence of disclosure information about unsupportive data on off-label prescribing decisions.

**Methods:**

Semi-structured interviews were conducted between December 2019 and January 2020 with oncologists (*n* = 35) and primary care physicians (*n* = 35). Interviews explored general prescribing practices, understanding of and information sources for learning about off-label use of prescription drugs, awareness of unsupportive data related to off-label uses, and preferences and reactions to disclosure statements about the existence of unsupportive data related to an off-label use.

**Results:**

Most participants reported prescribing drugs for off-label uses (with half reporting regular off-label prescribing). However, among those who prescribe off-label, approximately two-thirds had never seen unsupportive data about off-label uses. Physicians preferred a disclosure statement that provided a summary of the unsupportive data about the off-label use; this statement also led most physicians to say they were unlikely or less likely to prescribe the drug for that use.

**Conclusions:**

This study suggests that physicians’ decision-making about prescribing for off-label uses of approved drugs may be influenced by awareness of unsupportive data. Our interviews also suggest that providing more information about unsupportive study findings may result in a reduction in reported prescribing likelihood.

**Supplementary Information:**

The online version contains supplementary material available at 10.1186/s12875-022-01666-2.

## Introduction

Any uses of approved prescription drugs that are not included in the product labeling are considered unapproved, or off-label, uses [1]. These uses are not uncommon among physicians in certain medical specialties. Research with office-based physicians estimates that about 21% of all prescriptions are for off-label uses [2], with certain clinical specialties having much higher rates. For example, it is estimated that 50% to 75% of cancer therapies are used off-label [3]. Similarly, a large outpatient study found that 62% of pediatric visits resulted in an off-label prescription [4]. Other patient groups commonly prescribed off-label uses are psychiatric and critical care patients [5], as well as those with rare diseases that are treated by orphan drugs [6].

Despite the frequency of off-label prescribing, there are risks inherent to this practice. Off-label uses of approved products may lack sufficient data to support their use and have not passed the high evidence standards associated with U.S. Food and Drug Administration (FDA) approval [2, 7]. In fact, one study showed that over 70% of off-label uses lack evidence of efficacy [1]. Several other studies have illustrated adverse events resulting from off-label uses [8–10]. Under these circumstances, benefit-risk ratios for patients may not be well-established for off-label uses, and patients will bear the risks that could result from the unapproved use [7, 11]. Off-label prescribing also presents other non-health related risks that should be considered. Certain drugs that are often prescribed off-label, such as biopharmaceuticals, can be extremely costly and may not be covered by a patient’s insurance [3]. In addition, there is a possibility of legal risk to the physician, particularly when the off-label use is not the standard of care [5].

Physician awareness of the full scope of evidence related to off-label uses may help avoid or reduce adverse outcomes or other unintended consequences that can result. Available evidence about an off-label use – both supportive and unsupportive – can influence prescribers’ decisions about a drug’s appropriateness for a particular case [12]. The quality of studies for off-label use, however, varies widely [11, 13], and physicians do not always have sufficient research or statistical knowledge to critically assess the information [14, 15]. In addition, busy physician schedules [16, 17] make it difficult for them to find the time to seek additional information about these off-label uses.

Although studies do suggest that evidence about off-label uses can impact prescribing decisions (as noted above), there is little data to show whether physicians are typically *aware* of evidence related to off-label uses. Similarly, there are few studies that provide guidance on *how much* and *what type* of information could influence or change a prescribing decision.

With these research gaps in mind, we designed a survey-based experimental study that focuses on the impact of communicating unsupportive data related to off-label uses to physicians. Making physicians aware of the existence of unsupportive data may influence their perception of off-label uses and their prescribing decisions. Due to the limited existing research on these topics, however, we chose to first gather information using a structured qualitative method to inform the development of subsequent quantitative research. Thus, the overarching goal of this qualitative pre-study was to collect data to support the design of the experimental study, stimuli, and survey questions. The qualitative study’s two specific objectives, which are the focus of this paper, were: (1) to examine physician perceptions about off-label uses generally, including their awareness of unsupportive data; and (2) to explore the influence of disclosure information about unsupportive data on off-label prescribing decisions.

## Methods

### Design

We conducted a descriptive, qualitative study, using semi-structured interviews to explore the primary objectives of the study (see above). The research described in this paper was intended to inform a future experimental, quantitative phase of this project to assess various approaches to the disclosure of unsupportive data. For the current phase, we felt that qualitative interviews would provide the most robust and rich data on physician experiences to design the upcoming experimental study. Semi-structured physician interviews were conducted between December 2019 and January 2020. The Westat Institutional Review Board reviewed and approved the study.

### Participants

Our participants included practicing oncologists (*n* = 35) and primary care physicians (PCPs) (*n* = 35) who were recruited from opt-in research panels specializing in healthcare professionals. Members of research panels agree to periodically participate in surveys, interviews, and focus group studies. Panel participants receive rewards such as panel points and/or monetary incentives for their participation. We used a quota-based convenience sampling design for the current study, wherein interview recruitment was terminated once each physician group met their sample quota requirements (in this case, 35 physicians within each group).

Eligible physician participants were required to be American Medical Association-validated U.S. physicians, write 50 or more prescriptions per week, and spend at least half of their time in direct patient care. Physicians were excluded if they worked in marketing, advertising, or the pharmaceutical industry; if they worked for the U.S. Department of Health and Human Services; or if they had participated in a focus group or interview within the three months prior to the interview. Physicians were also required to have access to a computer and high speed internet for interviews in order to access the online meeting platform, WebEx.

### Disclosure Statements

Three unsupportive data disclosure statements (Table 1), providing varying levels of detail about unsupportive data, were reviewed in study interviews. The disclosure statements were included in a one-page brief report describing a study about the off-label use of a drug. The report was formatted to resemble a short journal article, and included one disclosure statement in its header. The brief report (with disclosure statement) was shared in WebEx during the interview for physicians to read at their own pace. Physicians successively reviewed all three statements, and presentation order was randomized across participants.

**Table 1:** Text of Disclosure Statements Reviewed by Physicians

**Disclosure Statement Text**	
1	*Not an Approved Indication* Use of topical imiquimod as a neoadjuvant treatment of lentigo maligna is not an approved indication based on the current imiquimod US Prescribing Information *Other Data* Another study that used topical imiquimod to treat LM found a significantly increased rate of recurrence (9.6% within three years of follow up), compared to other studies of topical imiquimod. In addition, ten percent of the study’s initial patient sample had to withdraw due to adverse reactions to topical imiquimod. (Spangelo BD, Grosz OJH, Joyce JM. Effect of topical imiquimod as primary treatment for lentigo maligna: Results from a phase II clinical trial. Arch Dermatol. 2012;148(7):928 930.)
2	*Not an Approved Indication* Use of topical imiquimod as a neoadjuvant treatment of lentigo maligna is not an approved indication based on the current imiquimod US Prescribing Information *Other Data* Another study investigating the use of topical imiquimod as a treatment strategy to prevent surgery for LM did not support the conclusion displayed here. (Spangelo BD, Grosz OJH, Joyce JM. Effect of topical imiquimod as primary treatment for lentigo maligna: Results from a phase II clinical trial. Arch Dermatol. 2012;148(7):928–930.)
3	*Not an Approved Indication* Use of topical imiquimod as a neoadjuvant treatment of lentigo maligna is not an approved indication based on the current imiquimod US Prescribing Information *Other Data* Other study results may not support the conclusion(s) displayed here

### Interview Guide

Interviewers used a semi-structured guide to explore general prescribing practices, understanding and learning about off-label use of prescription drugs, awareness of unsupportive data that related to off-label uses, and preferences and reactions to disclosure statements about the existence of unsupportive data related to an off-label use. Additional File 1 includes the complete interview guide.

### Data collection

A team of four experienced interviewers conducted all 70 physician interviews using WebEx. The length of each interview ranged from 30 to 60 min. Informed consent documents were sent to physicians in advance of the interview. At the beginning of the interview, the interviewer reviewed the informed consent document and asked physicians for verbal informed consent to participate in the study and to record the interview. Interviewers were trained on the interview guide to assure standardized data collection.

### Analysis

Audio recordings were independently transcribed verbatim and analyzed thematically. Transcripts were imported into QSR NVivo 11 for coding and analysis. Our approach to qualitative analysis closely aligns with Miles and Huberman’s [18] and Miller et al.’s [19] multistep models, comprising data reduction, data reassembly, and synthesis. After importing transcripts into the NVivo database, we created a set of a priori codes (mapped to the topics in the interview guide) to begin the data reduction process (see Additional File 2). These codes allowed our team to simultaneously review like content across all interviews. We then read and analyzed each interview transcript for common themes (e.g., use of journals as an information source, perception of a disclosure statement as “clear” or “confusing”), creating emergent codes to highlight new issues and add specificity and detail to our initial codes. The data reassembly process involved using query functions in the software to determine the relative prevalence of various themes, if sentiments leaned towards the favorable or unfavorable (when applicable), and whether and how codes varied between PCPs and oncologists. Synthesis, the third step in the process, involved multiple readings of the query results to determine patterns in the data that addressed our project objectives. We also looked at outliers to determine whether they were truly idiosyncratic or indicative of emerging sub-themes.

Two researchers coded interview transcripts. To ensure that coding was applied similarly across coders, a subset of transcripts (approximately twenty percent, eight from each physician group) were double-coded. Throughout this process, the coders met to discuss questions, reach consensus about the application of codes, and revise the codebook. Cohen’s kappa scores (and/or percentage agreement) were calculated to assess agreement among coders. Final kappa scores ranged from 0.70 to 1.0, and percentage agreement ranged from 81 to 100 percent. Once acceptable agreement (defined as a kappa score of 0.70 or higher, or percentage agreement of 75% or higher) was reached among coders, all remaining transcripts were coded by one coder.

SPSS Version 25 was used to analyze descriptive statistics for demographic and practice characteristics, and preferences and prescribing likelihood measures for disclosure statements.

## Results

We found few differences between PCPs and oncologists in our study, and the results below reflect combined physician groups, unless otherwise noted.

### Physician characteristics

Most physicians included in our sample were male, and represented White or Asian racial groups. The majority practiced in office-based settings and had been in practice for over 15 years. Demographic and practice characteristics of participating physicians are presented in Table 2.

**Table 2:** Physician participant characteristics

	**Overall %** ***N* = 70**
Gender	
Female	34% (*N* = 24)
Male	66% (*N* = 46)
Race	
White	46% (*N* = 32)
Black/African American	6% (*N* = 4)
Asian	37% (*N* = 26)
American Indian/Alaska Native	1% (*N* = 1)
Other	7% (*N* = 5)
Prefer not to answer	3% (*N* = 2)
Hispanic Origin	
Yes	4% (*N* = 3)
Practice Setting	
Office-based	66% (*N* = 46)
Clinic	19% (*N* = 13)
Inpatient	6% (*N* = 4)
Other	10% (*N* = 7)
Age	
Under 35	4% (*N* = 3)
35 to 45	27% (*N* = 19)
46 to 55	39% (*N* = 27)
56 to 65	14% (*N* = 10)
Over 66	16% (*N* = 11)
Years Practicing	
Less than 5	3% (N = 2)
5 to 14	24% (*N* = 17)
15 to 24	46% (*N* = 32)
Over 25	27% (*N* = 19)

### Off-label Prescribing Practices

The vast majority of physician participants (*n* = 63) reported prescribing drugs for off-label use. Approximately half of physicians reported prescribing off-label regularly (e.g., daily, weekly), while slightly over one-third prescribed in this manner infrequently (e.g., monthly, a few times per year). The seven participants who did not prescribe drugs for unapproved indications were split between PCPs and oncologists (4 PCPs, 3 oncologists). The primary reasons for not prescribing for unapproved indications were either because they were prohibited (i.e., their practice did not allow that type of prescribing), or they were concerned about the potential consequences of the unapproved use.

### Importance of Prescribing for Approved Indications

When asked about the importance of using FDA-approved medications for approved versus unapproved uses, most physicians provided mixed responses. Although participants acknowledged the importance of using a drug primarily for its approved indications – including that it was more likely to be approved by the patient’s insurance – they also acknowledged the potential benefit that these medications can bring for patients when used for unapproved uses.

“…I think it's important for physicians to be pretty discriminating about prescribing medicine. So it's important to me that it's approved for a specific indication. That said, it's standard of care to use some drugs that aren't specifically approved by the FDA for a specific indication. So I just think you need to know the difference between what standard of care, even if it's not FDA approved for that indication, and when you're just going rogue.”

Few physicians stated that it was not important to them whether a medication was used for approved or unapproved indications. For these physicians, generally as long as there was some evidence that the medication was effective for the indication, such as studies published in peer-reviewed journals, they would use it regardless. A small number of physicians stated unequivocally it was important to always use a medication for its approved indication.

### Information Sources for Off-Label Uses

Physicians reported learning about off-label uses from a variety of sources. The most commonly reported source of information was journals, which was mentioned by over half of all participants. Three other sources noted by almost half of all participants were colleagues, electronic sources (e.g., Up to Date, Epocrates), and professional conferences and meetings. Few notable differences were seen between PCPs and oncologists; however, nearly half of oncologists reported professional associations as an additional source of information about off-label uses (whereas professional associations were mentioned by only two PCP participants). Commonly mentioned professional associations included the American Society of Clinical Oncology (ASCO) and the American Society of Hematology (ASH).

### Awareness of and Opinions About Unsupportive Data

Approximately two-thirds of physicians who reported prescribing off-label stated they had never seen unsupportive data related to off-label uses, while only a small number were certain that they had. Among those who had seen such data but used the drug anyway, the most common justification for their decision was that the patient had no other treatment options.

Physicians were also asked, if they had an opportunity to learn about unsupportive data, what types of information would be important to review. The most common responses included:

- Details about study design

“So basically if there is controversy, you want to see the structure of the trial. What was the entry criteria, the patient types that they enrolled in each trial, let's say, if they're conflicting data. You cannot, as we say, compare apples to oranges.”

“The design ... Yeah, certainly the number of enrolled subjects, and whether it's a single-blind, a double-blind study, and if it's a double-blind study, whether it's cross-over. Study design certainly has a lot of bearing on the credibility.”

- Findings related to safety and side effects

“I would look for a clear picture of the toxicity to make a determination if the potential benefit outweighed the potential toxicity.”

“I'd like to know if there were side effects from the drug, what's the reason why they would not be indicating it for that particular use or that was it effective or the degree that it's effective and the degree of possible side effects and possible ill effects from using that drug. I'd like to have all that information.”

- Findings related to efficacy

“I look for evidence of efficacy that was corroborated in an independent source other than what the pharmaceutical rep gave me.”

“And really, just the effectiveness of the medication and was it found to be statistically significant or not?”

Most participants further noted that it was important for pharmaceutical companies to share the existence of unsupportive studies about off-label uses. When asked under what circumstances studies should be shared, physicians most often responded that any studies with differing results – whether or not statistically significant – should be shared.

“If we were going to disclose, then I think, disclosing anything that supported the opposite results or a neutral result, anything that's not in the same direction as the published study, would probably be the fairest way to do that.”

### Reactions to Disclosure Statements

The text of each disclosure statement included in our study is provided in Table 1. A summary of participant reactions is found below.

### Disclosure Statement 1

This statement noted that the described use was unapproved, and provided a summary of unsupportive findings and a citation for an unsupportive study. Physicians had generally positive reactions to this disclosure statement, describing it as helpful, informative, and clear. Several physicians also remarked that this statement caused them to look more critically at the data presented in the brief report. Several illustrative reactions to this statement are included below.

“I think it's great in this particular instance it clearly specifies that topical imiquimod is not an FDA approved therapy. Not only is there an absence of data of benefit, there's actual existence of data that may suggest harm.”

“Well, actually I think it's very clear verbiage and I think it's very neutral in terms of neither recommending or not recommending. But what's good is it's telling you what the use is that might be something that someone was interested in doing. So I think the verbiage is very clear. I would be happy to see a statement like that if I was looking for information.”

### Disclosure Statement 2

This statement noted that the described use was unapproved, and provided a statement that another study found unsupportive findings, along with a citation for the unsupportive study. Similar to disclosure statement 1, physicians had primarily positive feedback about this statement. Several illustrative reactions are found below.

“I think it's useful. The top part of it [is] well-phrased, that it's not approved. It's short and to the point and then it's good that it provides other data as an example.”

“…the disclosure is very transparent. They are telling what exactly they are going to talk about and the drug that they are mentioning that currently it's not approved but this has been our experience and there is conflicting data that does not support the conclusion that these investigators came to. It's very clear what they are trying to explain.”

### Disclosure Statement 3

This statement noted that the described use was unapproved, and mentioned that other studies may have found unsupportive findings. Physicians generally had negative reactions to disclosure statement 3, compared to the others reviewed. They noted that this statement was not helpful, that it seemed vague or incomplete, and that it was unclear and confusing. Illustrative reactions include:

“…this is incomplete with the other data part, right? It [says] other studies may not support the conclusions. I think that's very, it's written in a very gray zone kind of a way where it's not clear what they exactly mean to say. Does that mean that they are contradictory? Does that mean that it did not show the same result and what's the data right there? What's the reference? What's the reference that you're mentioning saying that there are other studies, those may not support the conclusions.”

“…the other data information is quite limited. It leaves me in a little bit of confusion or hesitation, at this time on topics mentioned that it's of course, it's not an approved indication. Then of course, it mentioned that there are other data studies which were done, and the study results are not what it is displayed here…This will leave me in a little bit of confusion at this time just with what is written there.”

### Disclosure Influence on Prescribing Likelihood

When asked about prescribing likelihood after reviewing the disclosure statements, all three statements led physicians to report they would be unlikely or less likely to prescribe the drug for an off-label use. Disclosure statement 1, which provided specific information about efficacy and safety findings that did not support off-label use, reduced reported prescribing likelihood the most. After viewing disclosure statement 1, over three-quarters of participants reported they would not or were less likely to prescribe the drug. Disclosure statement 3, which included a general statement that other data not supporting the use may exist, had the smallest impact on reported prescribing likelihood, with slightly fewer than half of participants reporting that they would not or were less likely to prescribe the drug after viewing the statement. 

### Disclosure Preferences

When asked which disclosure was preferred, most participants (*n* = 57) across the two physician groups preferred disclosure statement 1. This statement was preferred by 30 PCPs and 27 oncologists. Most physicians preferred this statement because it was perceived as more informative than the other statements.

“Because it gives more information, I don't have to go searching for why there was an issue, and if I think I agree or disagree with what the other data was, I can now have a way to quickly access it, and I know exactly what I'm looking for.”

“I like [it] better because it briefly summarizes why the other study does not support this conclusion. So I think for a busy clinician, you know they got the answer right away.”

Overall, 60 physician participants across the two groups selected disclosure statement 3 as their least preferred disclosure. Across the two groups, this statement was selected as least preferred by 32 PCPs and 28 oncologists. Disclosure statement 3 was seen as too vague by most physicians.

“Well, because it's kind of vague and it just says other study results may not support. So are there other studies and if they didn't support it, what are they? Who knows? I mean, at this institution it may have just been placebo effect and their patients may have been skewed and some other factor. Who knows?”

“Yeah, I mean that's probably the one that makes me sleep best at night in using an indication. It's like kind of don't ask, don't tell, so that's just kind of the most limited and no other studies may not support this conclusion. Okay. But I really don't know what the data is, as opposed to me being able to look directly at an alternative source of information and kind of have clinical information regarding occurrence rate and discontinuation rate.”

## Discussion

Data that support and conflict with off-label uses are often available to physicians. Despite access to such information, few physicians in our sample reported encountering unsupportive data about off-label uses. There are several possible explanations for this finding. Given time constraints and challenges in performing effective searches, it is unlikely physicians spend much time seeking information [20–22]. In addition, physicians’ confidence in their clinical judgement may lead them to view seeking information as unnecessary or merely confirming what they already know [21]. Publication biases favoring studies with significant results [23] may also contribute to lack of awareness of unsupportive data for off-label uses. Further, physicians may prescribe only a few drugs off-label for which supporting data exist and unsupportive data does not.

Our findings are important because they suggest that physicians’ decision-making about off-label uses of approved drugs – demonstrated in our study by reported prescribing likelihood – may be influenced by awareness of the existence of unsupportive data. Our results are consistent with research conducted by Schwartz and colleagues [24], which found that physicians provided information about an off-label use, along with evidence showing no clinical benefit for that use, were less likely to endorse the off-label use. Our interviews also suggest that providing more information about unsupportive study findings (as seen in disclosure statement 1 compared to statements 2 or 3) results in a more pronounced reduction in reported prescribing likelihood.

Our study has several limitations. First, the drug discussed in the brief report was in a specialty outside of normal practice for PCPs and oncologists. We selected this product intentionally to ensure that all interview participants approached the brief report with a similar level of knowledge, but a more familiar drug may have differentially affected behavioral intentions. Second, participants were primed to a discussion of off-label uses of drugs (due to the order of interview questions), and as such, may have been more cautious in their interpretations of the brief report and disclosure statement information. Third, because the study relied on qualitative methods, and probes were tailored to participant responses, there was some variation in data collection. Fourth, we were only able to investigate one specific example of unsupportive data, which contradicted both efficacy and safety findings. There may be a different outcome if the unsupportive data only contradict a safety finding or only find a difference in efficacy. Finally, our study used a convenience sample, and thus findings are not generalizable to the populations of U.S. PCPs or oncologists.

## Conclusion

Although some organizations have published best practices around the off-label use of medications [25], ultimately, decisions about off-label prescribing are in the hands of the physician. Having access to more complete information, including unsupportive information, about off-label uses of prescription drugs may change or otherwise impact a physician’s prescribing decision. This study provides important information about physician awareness of unsupportive data, as well as its potential impact on prescribing decisions. Findings from our qualitative study suggest that there is limited awareness of, and exposure to, unsupportive data about off-label uses among physicians. It is unclear whether this is due to a lack of published studies containing unsupportive data, a lack of information seeking, or other reasons. Regardless of the reason, it appears that the information physicians receive about off-label use tends to support that use, so finding ways to increase awareness of existing unsupportive data may be beneficial. Our study put forward one option, a relatively short disclosure statement that provides some brief context about the existence of unsupportive data, and tested three versions of that statement that included varying amounts of information. Although more research is needed, particularly regarding the ideal amount of information to provide, our study suggests that even a brief statement about unsupportive data may influence prescribing decisions. Using the information we gathered in this qualitative study, we will continue to explore this topic in a subsequent experimental study that will test the impact of disclosure statements with a larger sample of physicians.

Further studies could also examine nuances within the safety and efficacy of the unsupportive data or different levels of evidence supporting that data. Although some research shows physicians can correctly identify low methodological study rigor [26], other research suggests that they are not good at determining if data are of high enough quality to support a clinical decision [27, 28]. It would be useful to explore physicians’ ability to identify and understand poor quality unsupportive data, and the influence these factors have on their prescribing behaviors. More research is also needed to better understand the ideal amount of detail to provide, and reasons why physicians are not typically aware of unsupportive data.

## Supplementary Information

**Additional file 1.** Interview Guide

**Additional file 2.** Codebook

## Data Availability

The datasets generated and/or analyzed during the current study are not publicly available due to assurances of confidentiality made during the informed consent process. Anonymized and de-identified data may be requested from the corresponding author.
