# Supplementary material for: Influence of data disclosures on physician decisions about off-label uses: findings from a qualitative study
Source: BMC Prim Care. 2022 Apr 19;23:87. doi: 10.1186/s12875-022-01666-2 (PMC9017050; doi:10.1186/s12875-022-01666-2)
Supplement: Supplementary file 1 — Additional file 1. Interview Guide [file 12875_2022_1666_MOESM1_ESM.docx]

**Interview Guide**

**A. Background Information**

I’d like to begin with a few questions about your prescribing habits.

1. How often do you prescribe a drug you’ve never prescribed before?

2. What sources of information do you use to learn about a drug you’ve never prescribed before?

**B. Understanding and Learning about Unapproved New Uses**

3. As you know, prescription drugs are approved by FDA for one or more specific indications. However, they are sometimes *prescribed* for indications that were not approved by FDA. There are different terms for this, including “unapproved new use,” “unapproved use,” and “off-label use.” Which of these terms, if any, do you generally use? Why?

PROBE: Is there another term that you prefer instead of these? If so, what is it?

PROBE: For you, what differences, if any, are there between these terms?

*INTERVIEWER: NOTE RESPONDENT’S PREFERRED TERM AND USE IT FOR REMAINDER OF INTERVIEW IN PLACE OF [UNAPPROVED/OFF-LABEL/OTHER].*

4. What sources do you use to learn about [*unapproved/off-label/other*] uses of drugs?

PROBE: [*IF ONLY ONE SOURCE IS MENTIONED*] What other sources would you consider using?

PROBE: Which sources do you find most credible?

5. How can you tell when a drug’s indication is [*unapproved/off-label/other*]?

PROBE: Is it ever unclear whether the described indication is approved or unapproved? If so, what additional information would help make this clear?

6. Once a drug has been approved by FDA, how important is it to you whether the drug is used for an indication that is approved or unapproved?

**C. Understanding/Awareness of Contrary Data**

7. How often do you prescribe drugs for [*unapproved/off-label/other*] uses?

*INTERVIEWER: IF RESPONDENT PROVIDES AN ANSWER INDICATING THAT HE/SHE PRESCRIBES DRUGS OFF-LABEL, SKIP TO Q8. IF RESPONDENT ANSWERS “NEVER,”PROCEED TO Q7A.*

7a. Please tell me a little about why you don’t write [*unapproved/off-label/other*] prescriptions.

*INTERVIEWER: SKIP TO Q11.*

8. Can you give me an example of a drug that you prescribe for an [*unapproved/off-label/other*] use, and what condition you prescribe it for?

*INTERVIEWER: NOTE DRUG AND USE MENTIONED IN Q8.*

9. How did you learn about that use for [*Q8 drug*]?

10. Have you ever seen any conflicting or contrary information about the [*unapproved/off-label/other*] use, such as data suggesting that [*Q8 drug*] is *not* effective for [*Q8 use*] or had more risks than the approved use? If so, how did you learn that information?

11. Imagine a pharmaceutical company has shared a publication describing an [*unapproved/off-label/other*] use of one of their medications. Other studies may also exist that have conflicting or contrary findings from the study being shared by the pharmaceutical company. Thinking about the studies that have conflicting or contrary findings, what information would you want to know about them?

PROBE: What level of detail would you want?

PROBE: What do you think is the best way to provide information to prescribers about conflicting or contrary study findings?

**D. Discussion of Potential Disclosures**

Now I’m going to ask you review some example brief reports related to [*unapproved/off-label/other*] uses of a drug. Please read the sample that I put on the screen. After you’ve finished, I’ll ask you a few follow-up questions about what you read.

*INTERVIEWER: SHOW FIRST CONDITION [2, 3, OR 4]. ROTATE ORDER OF WHICH IS PRESENTED FIRST, SECOND, AND THIRD.*

12. The brief report that you read includes an example of what a disclosure for an [*unapproved/off-label/other*] use might look like. What are some of your initial reactions to the disclosure statement at the top of the page?

13. If you were considering prescribing a medication for an [*unapproved/off-label/other*] use and saw a disclosure like this at the top, what would you do?

PROBE: What additional information, if any, would you want to see?

PROBE: How likely would you be to prescribe this medication, if you saw this disclosure?

PROBE: [*IF RESPONDENT WOULD NOT PRESCRIBE*] What about that statement would affect your decision?

Now let’s review this next one. The brief report is identical, so please focus on the disclosure statement.

*INTERVIEWER: SHOW SECOND CONDITION.*

14. What are some of your initial reactions to this disclosure statement at the top of the page?

PROBE: How does this compare with the previous one you reviewed?

15. If you were considering prescribing a medication for an [*unapproved/off-label/other*] use and saw a disclosure like this at the top, what would you do?

PROBE: What additional information, if any, would you want to see?

PROBE: How likely would you be to prescribe this medication, if you saw this disclosure?

PROBE: [*IF RESPONDENT WOULD NOT PRESCRIBE*] What about that statement would affect your decision?

Finally, let’s take a look at one more. Again, the brief report is the same, but the disclosure differs.

*INTERVIEWER: SHOW THIRD CONDITION.*

16. What are some of your initial reactions to this disclosure statement at the top of the page?

PROBE: How does this compare with the previous ones you reviewed?

17. If you were considering prescribing a medication for an [*unapproved/off-label/other*] use and saw a disclosure like this at the top, what would you do?

PROBE: What additional information, if any, would you want to see?

PROBE: How likely would you be to prescribe this medication, if you saw this disclosure?

PROBE: [*IF RESPONDENT WOULD NOT PRESCRIBE*] What about that statement would affect your decision?

*INTERVIEWER: SHOW ALL THREE DISCLOSURE STATEMENTS*.

Now, I’ve placed all three disclosure statements on screen.

18. Which of these statements do you like best? Please explain.

19. Which of these statements do you like least? Please explain.

20. What suggestions, if any, do you have for how a disclosure about conflicting study results should be worded?

PROBE: What suggestions do you have for the wording of the three disclosure statements you reviewed?

PROBE: How do you feel about the inclusion of a citation for the conflicting study?

PROBE: Can you think of other ways of presenting information from conflicting studies that might work better than the disclosures? If so, what would you prefer?

**E. Wrap-Up**

21. Have you previously read the study described in the brief report I showed you earlier?

22. In general, how different do the results of two studies need to be in order to warrant a disclosure statement like the examples you reviewed today?

23. How important is it to you that pharmaceutical companies share conflicting or contrary study findings with prescribers?

24. Do you have any other comments about how conflicting or contrary study results could be provided to prescribers?

Thank you for your time and your helpful comments.
