# Supplementary material for: Influence of data disclosures on physician decisions about off-label uses: findings from a qualitative study
Source: BMC Prim Care. 2022 Apr 19;23:87. doi: 10.1186/s12875-022-01666-2 (PMC9017050; doi:10.1186/s12875-022-01666-2)
Supplement: Supplementary file 2 — Additional file 2. Codebook [file 12875_2022_1666_MOESM2_ESM.docx]

| **Code** | **Definition** | **Notes** |
| --- | --- | --- |
| ***A2. What sources of information do you use to learn about a drug you’ve never prescribed before?*** | | |
| Q2_NEVERPRESCSOURCES_COLLEAGUES | Code if response to Q2 mentions learning about new drugs from **colleagues and other physicians**. |  |
| Q2_NEVERPRESCSOURCES_JOURNAL | Code if response to Q2 mentions learning about new drugs from **journal articles, studies, or clinical trials**. |  |
| Q2_NEVERPRESCSOURCES_CONF | Code if response to Q2 mentions learning about new drugs from **conferences or meetings**. | This would include educational sessions such as CME meetings or dinners. |
| Q2_NEVERPRESCSOURCES_ELEC | Code if response to Q2 mentions learning about new drugs from **electronic sources, including websites, emails, and apps such as Up To Date**. | Electronic sources includes mentions of “googling.” |
| Q2_NEVERPRESCSOURCES_PHARMA | Code if response to Q2 mentions learning about new drugs from **pharmaceutical companies, including by looking at package inserts or from pharmaceutical reps**. |  |
| Q2_NEVERPRESCSOURCES_PROFASSN | Code if response to Q2 mentions learning about new drugs from **professional organizations**, such as ASCO or NCCN. | This may be double-coded, if applicable. For instance, a mention of an ASCO conference should be coded here, as well as to Q2_NEVERPRESCSOURCES_CONF. |
| Q2_NEVERPRESCSOURCES_OTHER | Code if response to Q2 mentions learning about new drugs from **other sources** not mentioned in other codes. |  |
| ***B3. As you know, prescription drugs are approved by FDA for one or more specific indications. However, they are sometimes prescribed for indications that were not approved by FDA. There are different terms for this, including “unapproved new use,” “unapproved use,” and “off-label use.” Which of these terms, if any, do you generally use? Why?*** | | |
| Q3_TERMS_OFFLABEL | Code if response to Q3 mentions preference for or familiarity with the term "**off label**." |  |
| Q3_TERMS_OTHER | Code if response to Q3 mentions preference for **any other term besides "off label.**" |  |
| Q3_TERMS_NODIFFS | Code if response suggests that there are **no differences between the terms** "off label," "unapproved use," and "unapproved new use." | It is possible to code *both* Q3_TERMS_NODIFFS and Q3_TERMS_DIFFS – for example, if the respondent says they personally do not see any differences between terms but could see others perceiving differences. |
| Q3_TERMS_DIFFS | Code if response suggests that using the **term "unapproved" is more negative** than saying "off label," or that "off label" is a more positive term. | See note for Q3_TERMS_NODIFFS. |
| ***B4. What sources do you use to learn about [unapproved/off-label/other] uses of drugs?*** | | |
| Q4_SOURCES_COLLEAGUES | Code if response to Q4 mentions learning about off label uses from **colleagues and other physicians**. |  |
| Q4_SOURCES_JOURNAL | Code if response to Q4 mentions learning about off label uses from **journal articles, studies, or clinical trials**. |  |
| Q4_SOURCES_CONF | Code if response to Q4 mentions learning about off label uses from **conferences or meetings**. | This would include educational sessions such as CME meetings or dinners. |
| Q4_SOURCES_ELEC | Code if response to Q4 mentions learning about off label uses from **electronic sources, including websites, emails, and apps such as Up To Date**. | Electronic sources includes mentions of “googling.” |
| Q4_SOURCES_PHARMA | Code if response to Q4 mentions learning about off label uses from **pharmaceutical companies, including by looking at package inserts or from pharmaceutical reps**. |  |
| Q4_SOURCES_PROFASSN | Code if response to Q4 mentions learning about off label uses from **professional organizations**, such as ASCO or NCCN. | This may be double-coded, if applicable. For instance, a mention of an ASCO conference should be coded here, as well as to Q4_SOURCES_CONF. |
| Q4_SOURCES_OTHER | Code if response to Q4 mentions learning about off label uses from **other sources** not mentioned in other codes. |  |
| ***B5. How can you tell when a drug’s indication is [unapproved/off-label/other]?*** | | |
| Q5_HOWTELLOFFLABEL_LOOKUP | Code if respondent indicates they will **look up approved indications**, using online sources, package inserts, Up to Date, etc. |  |
| Q5_HOWTELLOFFLABEL_EXP | Code if respondent suggests they know from **past clinical experience**, including vague statements suggesting they "just know" whether indication is on or off label. |  |
| Q5_HOWTELLOFFLABEL_OTHER | Code **all other ways** that respondent tells whether an indication is off label. |  |
| ***B6. Once a drug has been approved by FDA, how important is it to you whether the drug is used for an indication that is approved or unapproved?*** | | |
| Q6_ONLABELIMP_YES | Code if response to Q6 indicates it **is important** that drug is used for approved indication. | Responses should be coded to *only one* of the four codes under B6. |
| Q6_ONLABELIMP_NO | Code if response to Q6 indicates it **is not important** that drug is used for approved indication. | Responses should be coded to *only one* of the four codes under B6. |
| Q6_ONLABELIMP_MIXED | Code if response to Q6 is **mixed**, with respondent providing reasons why it is and is not important that drug is used for approved indication. | Responses should be coded to *only one* of the four codes under B6. |
| Q6_ONLABELIMP_UNCLEAR | Code if response to Q6 is **unclear**. | Responses should be coded to *only one* of the four codes under B6. |
| ***C7. How often do you prescribe drugs for [unapproved/off-label/other] uses?*** | | |
| Q7_OFFLABELPRESC_REG | Code if response to Q7 indicates that they prescribe off label **regularly**. | “Regular” prescribing may also be described using words such as “common.” |
| Q7_OFFLABELPRESC_INFREQ | Code if response to Q7 indicates that they prescribe off label **infrequently**. |  |
| Q7_OFFLABELPRESC_NO | Code if response to Q7 indicates that **they never or very rarely prescribe off label**. |  |
| ***C9. How did you learn about that use for [Q8 drug]?*** | | |
| Q9_LEARNABOUTUSE_TRAINING | Code if response to Q9 mentions learning about the off label use as part of **physician training**, including medical school, residency, and fellowships. |  |
| Q9_LEARNABOUTUSE_COLLEAGUES | Code if response to Q9 mentions learning about the off label use from **colleagues and other physicians**. |  |
| Q9_LEARNABOUTUSE_JOURNAL | Code if response to Q9 mentions learning about the off label use from **journal articles, studies, or clinical trials**. |  |
| Q9_LEARNABOUTUSE_CONF | Code if response to Q9 mentions learning about the off label use from **conferences or meetings**. | This would include educational sessions such as CME meetings or dinners. |
| Q9_LEARNABOUTUSE_ELEC | Code if response to Q9 mentions learning about the off label use from **electronic sources, including websites, emails, and apps such as Up To Date**. | Electronic sources includes mentions of “googling.” |
| Q9_LEARNABOUTUSE_PHARMA | Code if response to Q9 mentions learning about the off label use from **pharmaceutical companies, including by looking at package inserts or from pharmaceutical reps**. |  |
| Q9_LEARNABOUTUSE_ PROFASSN | Code if response to Q9 mentions learning about the off label use from **professional organizations**, such as ASCO or NCCN. | This may be double-coded, if applicable. For instance, a mention of an ASCO conference should be coded here, as well as to Q9_LEARNABOUTUSE_CONF. |
| Q9_LEARNABOUTUSE_OTHER | Code if response to Q9 mentions learning about the off label use from **other sources** not mentioned in other codes. |  |
| ***C10. Have you ever seen any conflicting or contrary information about the [unapproved/off-label/other] use, such as data suggesting that [Q8 drug] is not effective for [Q8 use] or had more risks than the approved use? If so, how did you learn that information?*** | | |
| Q10_SEENCONTDATA_NO | Code if respondent said **they had never seen any contrary data** about an off label use. | Responses should be coded to *only one* of the three codes under C10. |
| Q10_SEENCONTDATA_YES | Code if respondent said **they had seen contrary data** about an off label use. | Responses should be coded to *only one* of the three codes under C10. |
| Q10_SEENCONTDATA_OTHER | Code if the response is **unclear or does not seem to answer the question**. | Responses should be coded to *only one* of the three codes under C10. |
| ***C11. Imagine a pharmaceutical company has shared a publication describing an [unapproved/off-label/other] use of one of their medications. Other studies may also exist that have conflicting or contrary findings from the study being shared by the pharmaceutical company. Thinking about the studies that have conflicting or contrary findings, what information would you want to know about them?*** | | |
| Q11_INFOABOUTCONTRARYSTUDY_SPONSOR | Code if respondent wants to know **who sponsored the study**. |  |
| Q11_INFOABOUTCONTRARYSTUDY_DESIGN | Code if respondent wants to know about **study design and/or sample** (e.g., trial type, endpoints, sample size, demographics, etc.) |  |
| Q11_INFOABOUTCONTRARYSTUDY_EFFICACY | Code if respondent wants **efficacy or effectiveness data**. |  |
| Q11_INFOABOUTCONTRARYSTUDY_SAFETY | Code if respondent wants **safety data** (e.g., adverse events, side effects, etc.). |  |
| ***D12. The brief report that you read includes an example of what a disclosure for an [unapproved/off-label/other] use might look like. What are some of your initial reactions to the disclosure statement at the top of the page?*** | | |
| Q12_D1REACTION_HELPFUL | Code if response suggests that the disclosure statement is seen as **helpful, useful, or informative**. | This code should also be used when respondent suggests the disclosure is *more helpful/useful/etc*., compared to other disclosures. |
| Q12_D1REACTION_NOTHELPFUL | Code if response suggests that the disclosure statement is seen as **not helpful, not useful, or not informative**. | This code should also be used when respondent suggests the disclosure is *less helpful/useful/etc*., compared to other disclosures. |
| Q12_D1REACTION_VAGUE | Code if response suggests that the disclosure statement is seen as **vague, incomplete, or too brief**. |  |
| Q12_D1REACTION_SKEPTICISM | Code if respondent notes that the disclosure statement leads them to be **skeptical or cautious** of the off-label use. |  |
| Q12_D1REACTION_CLEAR | Code if response suggests that the disclosure statement is seen as **clear or direct**. | This code should also be used when respondent suggests the disclosure is *more clear*, compared to other disclosures. |
| Q12_D1REACTION_UNCLEAR | Code if response suggests that the disclosure statement is seen as **unclear or confusing**. | This code should also be used when respondent suggests the disclosure is *less clear*, compared to other disclosures. |
| Q12_D1REACTION_OTHER | Code if response is **neutral, does not suggest a clear opinion**, **or does not fit into other codes.** |  |
| ***D13. If you were considering prescribing a medication for an [unapproved/off-label/other] use and saw a disclosure like this at the top, what would you do?*** | | |
| Q13_D1WHATNEXT_NORX | Code if respondent **would not prescribe or would be less likely to prescribe**. | Each transcript should receive one *and only one* of the first three codes under D15. |
| Q13_D1WHATNEXT_YESRX | Code if respondent **would prescribe or would be more likely to prescribe**. | Each transcript should receive one *and only one* of the first three codes under D15. |
| Q13_D1WHATNEXT_OTHERRX | Code if respondent's prescribing decision is **not a clear yes or no,** or if the **disclosure would not impact the prescribing decision**. | Each transcript should receive one *and only one* of the first three codes under D15.  Use this code if the respondent indicates that the decision is context-dependent or uses language like “it depends.” |
| Q13_D1WHATNEXT_READCITATION | Code if respondent would **read the study** provided in the disclosure’s citation. | This code and all D13 codes below relate to additional information requested by respondents. If a respondent does not mention needing additional information, none of these codes will be used. |
| Q13_D1WHATNEXT_OTHERSTUDIES | Code if respondent would like to see **other studies** of the same indication. | See note in Q13_D1WHATNEXT_READCITATION.  This may include mentions of the respondent doing their own literature searches. |
| Q13_D1WHATNEXT_DESIGN | Code if respondent would like **study design details.** | See note in Q13_D1WHATNEXT_READCITATION.  This would include design details (trial type, endpoints, etc.) as well as study sample details. |
| Q13_D1WHATNEXT_SAFETY | Code if respondent would like more **details on safety or adverse reactions.** | See note in Q13_D1WHATNEXT_READCITATION. |
| Q13_D1WHATNEXT_FINDINGS | Code if respondent would like to see **study findings.** | See note in Q13_D1WHATNEXT_READCITATION.  This code is relevant because only one condition (2) presents a summary of study findings. For the other conditions, respondents may request a summary of study findings. |
| Q13_D1WHATNEXT_OTHERINFO | Code if respondent requests **additional information not covered in other codes**. | See note in Q13_D1WHATNEXT_READCITATION. |
| ***D14. What are some of your initial reactions to this disclosure statement at the top of the page?*** | | |
| Q14_D2REACTION_HELPFUL | Code if response suggests that the disclosure statement is seen as **helpful, useful, or informative**. | This code should also be used when respondent suggests the disclosure is *more helpful/useful/etc*., compared to other disclosures. |
| Q14_D2REACTION_NOTHELPFUL | Code if response suggests that the disclosure statement is seen as **not helpful, not useful, or not informative**. | This code should also be used when respondent suggests the disclosure is *less helpful/useful/etc*., compared to other disclosures. |
| Q14_D2REACTION_VAGUE | Code if response suggests that the disclosure statement is seen as **vague, incomplete, or too brief**. |  |
| Q14_D2REACTION_SKEPTICISM | Code if respondent notes that the disclosure statement leads them to be **skeptical or cautious** of the off-label use. |  |
| Q14_D2REACTION_CLEAR | Code if response suggests that the disclosure statement is seen as **clear or direct**. | This code should also be used when respondent suggests the disclosure is *more clear*, compared to other disclosures. |
| Q14_D2REACTION_UNCLEAR | Code if response suggests that the disclosure statement is seen as **unclear or confusing**. | This code should also be used when respondent suggests the disclosure is *less clear*, compared to other disclosures. |
| Q14_D2REACTION_OTHER | Code if response is **neutral, does not suggest a clear opinion**, **or does not fit into other codes.** |  |
| ***D15. If you were considering prescribing a medication for an [unapproved/off-label/other] use and saw a disclosure like this at the top, what would you do?*** | | |
| Q15_D2WHATNEXT_NORX | Code if respondent **would not prescribe or would be less likely to prescribe**. | Each transcript should receive one *and only one* of the first three codes under D15. |
| Q15_D2WHATNEXT_YESRX | Code if respondent **would prescribe or would be more likely to prescribe**. | Each transcript should receive one *and only one* of the first three codes under D15. |
| Q15_D2WHATNEXT_OTHERRX | Code if respondent's prescribing decision is **not a clear yes or no,** or if the **disclosure would not impact the prescribing decision**. | Each transcript should receive one *and only one* of the first three codes under D15.  Use this code if the respondent indicates that the decision is context-dependent or uses language like “it depends.” |
| Q15_D2WHATNEXT_READCITATION | Code if respondent would **read the study** provided in the disclosure’s citation. | This code and all D15 codes below relate to additional information requested by respondents. If a respondent does not mention needing additional information, none of these codes will be used. |
| Q15_D2WHATNEXT_OTHERSTUDIES | Code if respondent would like to see **other studies** of the same indication. | See note in Q15_D2WHATNEXT_READCITATION.  This may include mentions of the respondent doing their own literature searches. |
| Q15_D2WHATNEXT_DESIGN | Code if respondent would like **study design details.** | See note in Q15_D2WHATNEXT_READCITATION.  This would include design details (trial type, endpoints, etc.) as well as study sample details. |
| Q15_D2WHATNEXT_SAFETY | Code if respondent would like more **details on safety or adverse reactions.** | See note in Q15_D2WHATNEXT_READCITATION. |
| Q15_D2WHATNEXT_FINDINGS | Code if respondent would like to see **study findings.** | See note in Q15_D2WHATNEXT_READCITATION.  This code is relevant because only one condition (2) presents a summary of study findings. For the other conditions, respondents may request a summary of study findings. |
| Q15_D2WHATNEXT_OTHERINFO | Code if respondent requests **additional information not covered in other codes**. | See note in Q15_D2WHATNEXT_READCITATION. |
| ***D16. What are some of your initial reactions to this disclosure statement at the top of the page?*** | | |
| Q16_D3REACTION_HELPFUL | Code if response suggests that the disclosure statement is seen as **helpful, useful, or informative**. | This code should also be used when respondent suggests the disclosure is *more helpful/useful/etc*., compared to other disclosures. |
| Q16_D3REACTION_NOTHELPFUL | Code if response suggests that the disclosure statement is seen as **not helpful, not useful, or not informative**. | This code should also be used when respondent suggests the disclosure is *less helpful/useful/etc*., compared to other disclosures. |
| Q16_D3REACTION_VAGUE | Code if response suggests that the disclosure statement is seen as **vague, incomplete, or too brief**. |  |
| Q16_D3REACTION_SKEPTICISM | Code if respondent notes that the disclosure statement leads them to be **skeptical or cautious** of the off-label use. |  |
| Q16_D3REACTION_CLEAR | Code if response suggests that the disclosure statement is seen as **clear or direct**. | This code should also be used when respondent suggests the disclosure is *more clear*, compared to other disclosures. |
| Q16_D3REACTION_UNCLEAR | Code if response suggests that the disclosure statement is seen as **unclear or confusing**. | This code should also be used when respondent suggests the disclosure is *less clear*, compared to other disclosures. |
| Q16_D3REACTION_OTHER | Code if response is **neutral, does not suggest a clear opinion**, **or does not fit into other codes.** |  |
| ***D17. If you were considering prescribing a medication for an [unapproved/off-label/other] use and saw a disclosure like this at the top, what would you do?*** | | |
| Q17_D3WHATNEXT_NORX | Code if respondent **would not prescribe or would be less likely to prescribe**. | Each transcript should receive one *and only one* of the first three codes under D17. |
| Q17_D3WHATNEXT_YESRX | Code if respondent **would prescribe or would be more likely to prescribe**. | Each transcript should receive one *and only one* of the first three codes under D17. |
| Q17_D3WHATNEXT_OTHERRX | Code if respondent's prescribing decision is **not a clear yes or no,** or if the **disclosure would not impact the prescribing decision**. | Each transcript should receive one *and only one* of the first three codes under D17.  Use this code if the respondent indicates that the decision is context-dependent or uses language like “it depends.” |
| Q17_D3WHATNEXT_READCITATION | Code if respondent would **read the study** provided in the disclosure’s citation. | This code and all D17 codes below relate to additional information requested by respondents. If a respondent does not mention needing additional information, none of these codes will be used. |
| Q17_D3WHATNEXT_OTHERSTUDIES | Code if respondent would like to see **other studies** of the same indication. | See note in Q17_D3WHATNEXT_READCITATION.  This may include mentions of the respondent doing their own literature searches. |
| Q17_D3WHATNEXT_DESIGN | Code if respondent would like **study design details.** | See note in Q17_D3WHATNEXT_READCITATION.  This would include design details (trial type, endpoints, etc.) as well as study sample details. |
| Q17_D3WHATNEXT_SAFETY | Code if respondent would like more **details on safety or adverse reactions.** | See note in Q17_D3WHATNEXT_READCITATION. |
| Q17_D3WHATNEXT_FINDINGS | Code if respondent would like to see **study findings.** | See note in Q17_D3WHATNEXT_READCITATION.  This code is relevant because only one condition (2) presents a summary of study findings. For the other conditions, respondents may request a summary of study findings. |
| Q17_D3WHATNEXT_OTHERINFO | Code if respondent requests **additional information not covered in other codes**. | See note in Q17_D3WHATNEXT_READCITATION. |
| ***D18. Which of these statements do you like best? Please explain.*** | | |
| Q18_LIKEBEST_C2 | Code if respondent said they liked **Condition 2 best**. |  |
| Q18_LIKEBEST_C3 | Code if respondent said they liked **Condition 3 best**. |  |
| Q18_LIKEBEST_C4 | Code if respondent said they liked **Condition 4 best**. |  |
| Q18_LIKEBEST_OTHER | Code if respondent's most liked condition is **unclear**, if **multiple conditions are mentioned**, or if response **does not clearly fit** other codes. |  |
| ***D19. Which of these statements do you like least? Please explain.*** | | |
| Q18_LIKELEAST_C2 | Code if respondent said they liked **Condition 2 least**. |  |
| Q18_LIKELEAST_C3 | Code if respondent said they liked **Condition 3 least**. |  |
| Q18_LIKELEAST_C4 | Code if respondent said they liked **Condition 4 least.** |  |
| Q18_LIKELEAST_OTHER | Code if respondent's least liked condition is **unclear**, if **multiple conditions are mentioned**, or if response **does not clearly fit** other codes. |  |
| ***E22. In general, how different do the results of two studies need to be in order to warrant a disclosure statement like the examples you reviewed today?*** | | |
| Q22_HOWDIFF_ALLDIFFS | Code if response suggests that **any and all differences** should be disclosed, no matter now minor. | When respondent mentions statistical significance as a criteria, include it in this code.  Code *only one* of the first three codes under E22 – ALLDIFFS, MAJORDIFFS, and ALLDATA. |
| Q22_HOWDIFF_MAJORDIFFS | Code if response suggests that **major or clinically meaningful differences** should be disclosed. | Code *only one* of the first three codes under E22 – ALLDIFFS, MAJORDIFFS, and ALLDATA. |
| Q22_HOWDIFF_ALLDATA | Code if response suggests that **all other studies** should be disclosed, **whether they support or differ** from the study being presented by the pharmaceutical company. | Code *only one* of the first three codes under E22 – ALLDIFFS, MAJORDIFFS, and ALLDATA. |
| Q22_HOWDIFF_UNCERTAIN | Code if respondent suggests that they are uncertain about the answer to this question, or notes that context matters. | This response may be double-coded with the three codes listed above, but should not be double-coded with the “other” code below. |
| Q22_HOWDIFF_OTHER | Code if response is **unclear or does not clearly fit** into other codes. | Only use this code when the response does not fit it any other codes under E22. |
